# Patulin Mycotoxin in Mango and Orange Fruits, Juices, Pulps, and Jams Marketed in Pakistan

**DOI:** 10.3390/toxins12010052

**Published:** 2020-01-16

**Authors:** Shabbir Hussain, Muhammad Rafique Asi, Mazhar Iqbal, Nisha Khalid, Syed Wajih-ul-Hassan, Agustín Ariño

**Affiliations:** 1 Food Toxicology Laboratory, Nuclear Institute for Agriculture and Biology College (NIAB-C), Pakistan Institute of Engineering and Applied Sciences (PIEAS), Jhang Road, Faisalabad 38000, Pakistan; shabbir.ne@gmail.com (S.H.); nishakhalid22@gmail.com (N.K.); wajih599@yahoo.com (S.W.-u.-H.); 2 Central Analytical Facility Division, Pakistan Institute of Nuclear Science and Technology (PINSTECH), P. O. Nilore, Islamabad 45650, Pakistan; 3 Health Biotechnology Division, National Institute for Biotechnology and Genetic Engineering College (NIBGE-C), Pakistan Institute of Engineering and Applied Sciences (PIEAS), Jhang Road, Faisalabad 38000, Pakistan; 4 Instituto Agroalimentario de Aragón—IA2 (Universidad de Zaragoza-CITA), Facultad de Veterinaria, 50013 Zaragoza, Spain; aarino@unizar.es

**Keywords:** patulin, mango, orange, fruit-derived products, food safety, regulatory limits

## Abstract

The objective of the study was to explore the incidence of patulin (PAT) mycotoxin in mango and orange fruits and derived products marketed in Pakistan. A total of 274 samples, including 70 mango fruits, 63 mango-based products (juices, pulp, and jam), 77 orange fruits, and 64 orange-based products, were collected. PAT was determined by reverse-phase high-performance liquid chromatography (HPLC) with UV-Vis detector (276 nm). Linear detector response was observed (R^2^ > 0.99), the limit of detection (LOD) was 5 µg/kg and recovery percentage was 97.4%. The incidence of PAT in mango samples was 61.7%, and the concentration ranged from <LOD to 6415 µg/kg with a mean of 110.9 µg/kg. Our results showed the high susceptibility of mango fruits to patulin, and it was observed that decayed mango fruits were most contaminated with PAT. Among the mango samples, PAT concentration was higher in fruits than in processed products such as mango juice, pulp, and jam. Toxin incidence in orange samples was 52.5% with concentrations from <LOD to 61 µg/kg and a mean of 6.3 µg/kg. As much as 29 samples of mango (21.8%) contained PAT concentration above the regulatory limit (50 µg/kg), whereas there was only one exceeding orange sample (0.7%). Our results show that PAT seems to be a problem in fruits, juices, and derived solid products, especially from mango, and needs surveillance on regular basis.

## 1. Introduction

**Figure 1:**
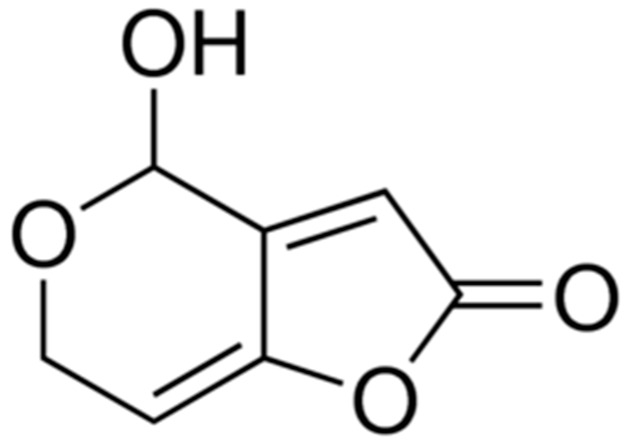
Molecular structure of patulin.

Mycotoxins are compounds produced by naturally occurring fungi having toxic nature for animals and humans [1,2,3]. It has been considered that approximately 25% of total world food crops annually are contaminated with mycotoxins [4]. Patulin mycotoxin (a polyketide lactone 4-hydroxy-4H-furo (3,2c) pyran-2 (6H)-one; Figure 1) [5,6] belongs to a class of toxic compound with low molecular weight (154.121 g/mol) [7,8]. The molecular formula of patulin (PAT) is C_7_H_6_O_4_; it is stable in aqueous media at 105–125 °C with melting point of 110 °C. It is a colorless and crystalline compound [9,10]. PAT is often associated with fruits, juices, and derived products, including foods intended for young children, because of the contamination with fungal species such as *Penicillium expansum*, *Aspergillus clavatus*, and *Byssochlamys nivea* [11]. These patulin-producing fungi attack susceptible products during growth, harvest, storage, or food processing. Among different fungi species, *Penicillium expansum*, which is commonly present in many varieties of fruits, is the major producer of PAT [12,13,14]. Patulin has been primarily associated with apple and apple-based products. However, the toxin may also contaminate other fruits, moldy feed, rotten vegetables, and wheat straw residue. It has been suggested that cold regions may become liable to temperate problems concerning patulin in foodstuffs due to climate change [15].

Due to contamination of food and feed at all phases of processing, storage, transportation, and sale, PAT has a critical effect in agriculture zone and food industry. PAT mycotoxin causes health hazards after ingestion of contaminated fruits and derived products. PAT toxicity relates to deleterious formation of adducts with sulfhydryl groups, producing acute and chronic toxicity problems in animals and humans [16]. Exposure to this mycotoxin is associated with immunological, neurological, and gastrointestinal outcomes such as distension, ulceration, and hemorrhage [17,18]. Body organs affected by PAT include kidney, liver, intestine, spleen, and stomach. PAT toxicity in mammalian cells and animals includes genotoxicity, teratogenicity, embryotoxicity, and immunotoxicity [19,20]. According to the International Agency for Research on Cancer (IARC), PAT is classified in the group 3 as “not classifiable as to its carcinogenicity to humans” [20].

The adverse health effects of PAT have led to the establishment of safe levels of PAT in foodstuffs. The Codex Alimentarius established the maximum level of PAT in fruits and juices at 50 µg/kg [21]. According to Commission Regulation (EC) No. 1881/2006, the European Union (EU) fixed maximum levels of PAT in fruit juices (50 µg/kg), solid apple products (25 µg/kg), and foods intended for infants and young children (10 µg/kg) [22]. Countries such as China, USA, and Canada have also established maximum levels for PAT in foods, primarily in apple-based products, in the range between 25 and 50 µg/kg [23,24,25]. Furthermore, the Joint Expert Committee for Food Additives has established a provisional maximum tolerable daily intake of 0.4 µg/kg body weight [26].

It is well established that the main sources of PAT in human diet are apples and apple-derived products, so the majority of reported studies concern patulin determination in apple-based foodstuffs [27,28]. However, monitoring of PAT in other fruits and fruit-derived products should not be neglected. A previous study carried out in Pakistan on various fruits, juices and smoothies showed the presence of PAT in more than 50% of samples with a mean concentration of 182 µg/kg (Iqbal et al., 2018) [29]. However, mango and orange fruits, and their derived products were not included in the survey.

In view of the above details, the present research has focused on exploring the current occurrence of PAT in mango and orange fruits, fruit juices and derived products, and to compare the levels of PAT with maximum regulatory levels.

## 2. Results

### 2.1. Method Validation

The patulin contamination in fruits and fruit-based products is a worldwide problem, and effective control of PAT strongly depends on reliable analytical methods. The validation of the analytical method for PAT included determination of linearity, recovery, precision (repeatability and reproducibility), and sensitivity (limit of detection, LOD, and limit of quantification, LOQ). Linearity was checked by injection into HPLC-UV of PAT standards in the range from 5 to 100 µg/L (Table 1), the correlation coefficient obtained was 0.9916. The average retention time of PAT was 6.383 ± 0.05 min with good coefficient of variation (0.75%). Recovery experiments were done by spiking negative samples of mango and orange at PAT concentrations of 10, 50, and 100 µg/kg. After 1 h, the spiked samples were processed and analyzed by HPLC. The average recovery was 97.4%, with good values for repeatability (relative standard deviation, RSD_r_ less than 5%) and reproducibility (RSD_R_ less than 15%). The limit of detection (LOD) and quantification (LOQ) were determined by signal-to-noise ratio and were 5 µg/kg and 15 µg/kg, respectively. In conclusion, the analytical method used allowed for accurate quantitative determination of patulin in mango and orange samples and fulfilled performance requirements of Commission Regulation (EC) No. 401/2006 [30].

**Figure 2:**
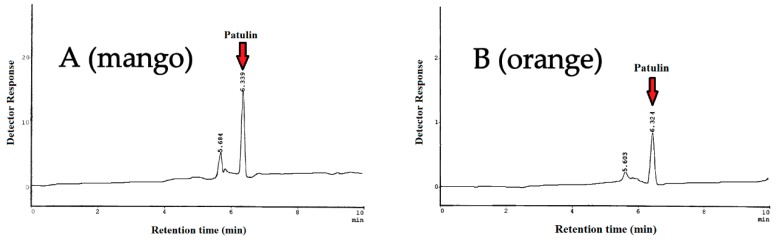
HPLC chromatograms of natural occurrence of patulin in mango sample (**A**) and orange sample (**B**).

The analytical method used is based on AOAC method 995.10, which was successfully validated through collaborative studies for patulin determination in apple products [31]. In detail, the method consists of four steps, including liquid–liquid extraction with ethyl acetate, sodium carbonate clean-up, sodium sulfate drying, and LC-UV determination. Na_2_CO_3_ neutralization is used to lower interference from the phenolic compounds in fruit matrices, such as the 5-hydroxymethylfurfural (5-HMF) [32]. The main shortcoming of the method is the presence of interfering matrix components that might affect chromatographic separation. To better remove interferences for patulin determination, a series of representative random samples were additionally subjected to a second purification step using multifunctional clean-up columns MFC 228. The chromatographic separation was improved, though no significant differences were observed in recovery percentage and patulin concentration. Figure 2 represents HPLC chromatograms of natural occurrence of patulin in mango and orange samples.

### 2.2. Occurrence of Patulin in Fruits and Derived Products

Results of PAT occurrence in 133 samples of mango fruits and derived products are shown in Table 2. A total of 70 samples of mango fruits along with 63 samples of mango-based products (juices, pulp, and jam) were randomly collected from different sites of Punjab, Pakistan. From the data, it is evident that 82 samples were found PAT-contaminated with an incidence level (61.7%) and a total mean concentration of 110.9 µg/kg. The percentage of positive samples of Faisalabad, Sheikhupura, Multan, Shorkot, and Rawalpindi was 50%, 33.3%, 53.3%, 71.4%, and 40%, respectively, whereas the percentage of contamination in mango juice, pulp, and jam was 75%, 87.5%, and 60%, respectively. The average PAT levels in mango fruits were 348 µg/kg, 42.6 µg/kg, 87.5 µg/kg, 254.2 µg/kg, and 14.7 µg/kg in samples collected from Faisalabad, Sheikhupura, Multan, Shorkot, and Rawalpindi, respectively. The average PAT levels in mango juices, pulp, and jam of different brands were 24.3 µg/kg, 82.3 µg/kg, and 5.0 µg/kg, respectively. Although the incidence of patulin was very similar between samples of mango fruit and derived products, the concentration was higher in the first (186.6 µg/kg) compared with the second (26.9 µg/kg). Among the mango fruit samples, it is noteworthy a sample from a Faisalabad’s local market with an extremely high PAT content (6415 µg/kg), as well as another sample from Shorkot with a very high PAT content of 2030 µg/kg. In the present study, healthy mango fruits were less contaminated with PAT in comparison with decayed ones.

Table 3 reports the PAT incidence and concentration in 141 samples of orange fruits and derived products, comprising 77 samples of orange fruits along with 64 samples of orange-based products (juices, pulp, and jam), which were randomly collected from different sites of Punjab, Pakistan. A total of 74 samples were found positive for PAT (52.5%) with a total mean concentration of 6.3 µg/kg, much lower than that of 110.9 µg/kg found in mango. The incidences in orange fruits were 60%, 88.9%, 28.6%, 53.9%, and 18.2% in samples taken from Faisalabad, Sargodha, Layyah, Toba Tek Singh, and Sahiwal, respectively, while 71.4%, 60%, and 21% was assessed in orange juices, pulp, and jams, respectively. The average concentration of PAT in orange fruits was 7.6 µg/kg, 8.1 µg/kg, 8.7 µg/kg, 5.1 µg/kg, and 1.6 µg/kg in samples from Faisalabad, Sargodha, Layyah, Toba Tek Singh, and Sahiwal, respectively. Additionally, orange juices, pulp, and jams contained 8.3 µg/kg, 6.5 µg/kg, and 1.1 µg/kg, respectively. Both incidence and levels of PAT were similar in orange fruits and derived products.

The percentage of mango samples that exceeded the maximum level of PAT (50 µg/kg) was 29 out of 133 (21.8%) (Table 2), while only 1 out 141 (0.7%) orange samples surpassed the maximum level (Table 3). The maximum percentage of violative mango samples came from Shorkot (57.1%), a city situated southwest of Faisalabad, while northern Rawalpindi, whose elevation above sea level is 508 m, showed the least noncompliant samples (10%). Similarly, exceeding orange samples only came from Layyah, a city located southwest of Faisalabad.

## 3. Discussion

The contamination with PAT in analyzed fruits and derived products showed higher incidence and concentration values in mango as compared with orange samples. Some mango samples exhibited PAT levels well above the regulatory limit (50 µg/kg), with a maximum of 6415 µg/kg in a sample from Faisalabad. Patulin-producing molds are responsible for the rotting of some fruits and vegetables, especially pomaceous fruits such as apples and pears [1]. Regarding mango samples, fruits that were decayed showed higher PAT levels as compared with apparently healthy fruits. In Pakistan, management by Good Agriculture Practices (GAPs) is not generally applied to mango production, and fungicides are not applied during the growth stage of mango fruits. In general, external wounds and ripening make fruits more sensitive to contamination by molds, so wounded and ripened fruits have a higher risk of contamination in the absence of fungicide use. Not in vain, extraordinarily high patulin levels up to 113343 µg/kg have been reported in the rotten area of an apple [33]. Therefore, wounded and ripened mangoes can be very exposed to postharvest diseases. To the best of our knowledge, there is no previous publication reporting the high susceptibility of mango to patulin-producing molds and the subsequent patulin contamination.

The extremely high level of patulin found in a mango sample (6415 µg/kg) is only comparable to those of 7339, 13808, and 19662 µg/kg reported in samples of fruit juices from Argentina [34]. Present results are also in agreement with a previous study carried out in Pakistan on patulin in different fruits, juices, and smoothies [29], with a maximum of 1100 µg/kg in a sample of red globe grapes. Other samples type such as seedless grapes, apples, pears, and tomato exceeded 500 µg/kg. Similarly, other authors have reported high patulin concentrations in apple juice from the USA (2700 µg/kg) [35] and concentrated juices from Tunisia (889 µg/kg) [2]. In Turkey, the maximum concentration of patulin in apple sour reached 1416 µg/kg [36]. In addition, fifty samples of apple juices were investigated for patulin levels in India [37], with an incidence of 24% positives and a maximum of 845 μg/kg. Beretta et al. analyzed 82 samples of apple-based foods for PAT, with maximum concentrations in juice made with apple pulp and in fruits. In rotten apples, not only was the amount of patulin very high in the rotten area (1150 μg/kg), but the mycotoxin had also spread to areas unaffected by fungus [38]. Finally, Yurdun et al. [39] reported a sample of apple juice with a patulin level of 733 μg/kg. In turn, PAT was not detected in mango juice from two studies carried out in Malaysia [40,41].

In other studies, patulin concentrations attained levels in the range of 5–50 μg/kg, such as Al-Hazmi et al. in samples of apple juice from Saudi Arabia [42]. In Greece, a study revealed the presence of PAT in 100% of the fruit juice samples examined [43]. The mean values of PAT in concentrated fruit juices and in commercial fruit juices were 10.54 μg/kg and 5.57 μg/kg, respectively. The most contaminated samples were four concentrated juices, ranging from 18.10 to 36.8 μg/kg. The mean concentration of patulin in orange juices was 6.80 μg/kg. In South Korea, a study on 72 samples of fruit juices reported nine positive for patulin (three apple, two orange, and four grape juices), with a maximum concentration of 30.9 μg/kg in an orange juice sample [44].

In the undertaken study, results on occurrence of PAT in mango and orange fruits and their derived products, which are consumed in Punjab, Pakistan, depicted higher contamination levels, especially from mango. PAT contamination in fruits and derived products is a burning issue for the health implications, so their surveillance is a basic and urgent need. In Pakistan, a variety of fruits with good flavor and taste are grown in tropical and subtropical climate and are available throughout the year. The various varieties of fruits are grown on an area of about 800,000 hectares with worth production of 7.05 million tones. During crop season 2017–2018, 10% of the total fruit production was exported [45]. Good agriculture practices (GAP) and postharvest control strategies must be adopted to inhibit PAT formation in fruits and derived products for the prevention and reduction of exposure to this mycotoxin. Proper picking, handling, and packaging operations, as well as storage and transportation of fruits can limit fungal growth and patulin production.

## 4. Conclusions

Patulin contamination in fruits and derived products is a worldwide problem due to its risky toxicity. An HPLC method with UV detection for the determination of PAT, which is fast, reliable, and sensitive, was successfully validated and could be applied for routine analysis and monitoring of fruits, pulps, juices, and derived products. PAT concentrations were higher in mango than in orange samples, and greater in whole fruits than in derived products such as juice, pulp, and jam. A significant percentage of mango samples exceeded the maximum levels established for patulin. Further studies are needed to develop strategies that are helpful to reduce the presence of patulin-producing fungi in these commodities. Therefore, a better understanding of the underlying mechanisms of patulin toxigenesis in mango is needed. Regular monitoring of fruits and their products during the harvest and processing stages is recommended to enhance the confidence of end users. The results of the present study would be highly beneficial for the horticulturists, processors, traders, and consumers.

## 5. Materials and Methods

### 5.1. Sampling

A total of 274 samples including 70 mango fruits, 40 mango juice, 8 mango pulp, 15 mango jam, 77 orange fruits, 35 orange juice, 10 orange pulp, and 19 orange jam were purchased in different areas of Central Punjab (Pakistan) from January to December 2018. The mango (*Mangifera indica*) and orange (*Citrus sinensis*) fruits were collected in supermarkets and local markets from Faisalabad, and from cities north (Rawalpindi, Sargodha, and Sheikhupura) or south (Multan, Shorkot, Layyah, Toba Tek Singh, and Sahiwal) Faisalabad. The mango- and orange-based products (juice, pulp, and jam) were from commercial brands of nationwide distribution purchased from supermarkets, local markets, and general stores in Faisalabad district. All samples were stored in their original packages at 4 ºC until they were analyzed. The samples were opened and thoroughly homogenized.

### 5.2. Chemicals and Reagents

Acetonitrile (HPLC grade), glacial acetic acid, sodium chloride, and sodium carbonate were purchased from Merck. Ethyl acetate, methanol, sodium sulfate anhydrous (analytical grade), 5-hydroximethyl furfural (5-HMF), and patulin (5 mg of solid crystalline) were purchased from Sigma-Aldrich (Saint-Louis, MO, USA). Stock solution of patulin 1 mg /mL was prepared in acetonitrile and stored at −4 °C. Necessary volumes of stock solution were taken for working solutions (5, 10, 30, 50, 70, 100 µg/L) in 0.1% acetic acid. PriboFast multifunctional cleanup columns MFC 228 (Pribolab Pte. Ltd., Singapore) were applied for the optimization of the purification process.

### 5.3. Sample Preparation and Extraction

The purchased fruit samples were chosen free from debris, washed with water, shade-dried, and cut into small pieces with sharp knife. A sample of about 500 g was homogenized using high-speed blender (Braun Blender Mix 2000, Marktheidenfel, Germany). Samples of products (juice, pulp, and jam) were taken directly from the original packages. Analytical method for PAT was based on AOAC method 995.10 with little modifications [31]. Homogenized samples (25 g or 25 mL in triplicate) were extracted twice with 50 mL ethyl acetate along with 2 g sodium chloride in 250 mL Erlenmeyer flask (Pyrex, Germany) by shaking at high speed in horizontal shaker (Gunther and Co, Bremen, Germany) for one hour. The upper organic layers were combined and cleaned up with sodium carbonate solution (1.5% Na_2_CO_3_). Cleaned extract was rapidly dehydrated with 10 g anhydrous sodium sulfate (Na_2_SO_4_) and filtered with Whatman No. 1 filter paper. For alternative second-time clean-up, 9 mL cleaned extract was passed through a multifunctional column MFC 228, and 4 mL purified extract was collected. The extracts were evaporated to dryness under a stream of nitrogen. The dry residue was immediately dissolved in 1000 µL of 0.1% acetic acid and passed through syringe filter (0.22 µm, Millipore, Darmstadt, Germany). The samples were analyzed for PAT by reverse-phase HPLC equipped with UV-Vis detector (SPD-10AS, Shimadzu, Japan) in isocratic mode. The injection volume was 20 µL, and total run time was 10 min for PAT analysis.

### 5.4. Apparatus and HPLC Conditions

**Figure 3:**
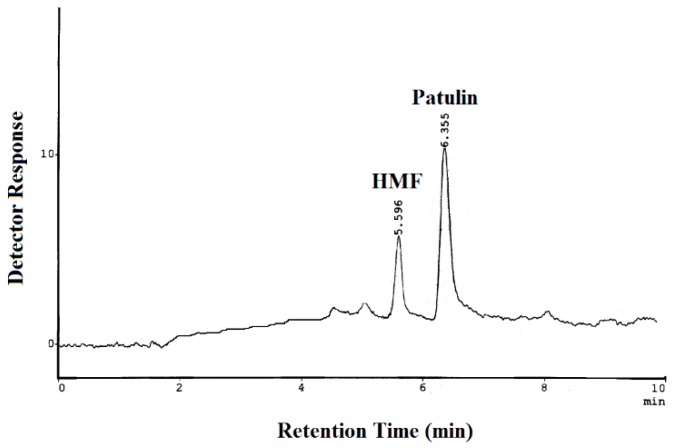
Chromatogram of 5-HMF (5-hydroximethyl furfural) and patulin standards.

HPLC comprised a delivery pump (LS-10AS), system controller (SCL-10A), column oven (CTO-10A), UV-Vis detector (SPD-10AS), and Communication Bus Module (CBM-101), Shimadzu, Japan. Separations were performed in a Discovery HS C18 silica-based column (250 × 4.6 mm, 5 µm particle size; Supelco, Bellefonte, PA, USA), maintained at 30 °C with a flow rate of 1.5 mL/min in isocratic mode. Mobile phase was a mixture of acetonitrile:water (10:90, *v/v*) filtrated with 0.45µm filter (Nylon 66 membranes filter, Supelco, Bellefonte, PA, USA). Cleaned sample extracts and standards were injected using a Rheodyne injector (20 µL loop) in reverse-phase system, and wavelength of detection was 276 nm. Chromatograms were received with CLASS LC-10 Acquisition software. Qualitative and quantitative determinations of PAT were made comparing the retention time and peak area of reference standards. For method validation, a test was performed to confirm the peak separation of PAT and its principal interference 5-HMF. A solution containing 2 µg/mL of 5-HMF and 2 µg/mL of PAT was prepared and injected into HPLC (Figure 3).

### 5.5. Statistical Analysis

The compiled data were subjected to statistical analysis. Triplicate results of PAT in fruits, juices, and derived products were calculated in Excel software, and data were given as mean with standard deviation. Samples with a concentration of PAT higher than the LOD were considered positive. For samples with a concentration below the LOD, a value of zero was considered for calculating the mean. The significance differences in PAT level between the groups were analyzed by one-way ANOVA test (*p* < 0.05) using SPSS (IBM SPSS Statistics 19, Armonk, NY, USA, 2010).

## Figures and Tables

**Table 1:** Linearity of standard working solutions of patulin (PAT). Values are mean of triplicate analysis.

Retention Time (min)	Concentration PAT µg/L	Average Peak Area (mV)	Standard Deviation	Coefficient of Variation (%)
6.383	5	327	16	4.89
6.430	10	533	21	3.94
6.381	30	1473	51	3.46
6.338	50	3451	129	3.74
6.444	70	4714	218	4.62
6.323	100	7181	283	3.94

**Table 2:** Incidence of patulin (µg/kg) in mango fruits and derived products.

Sample	Sampling Site	n Total (Positive)	Incidence %	Mean ± SD µg/kg	Maximum Value µg/kg	n (%) > 50 µg/kg
Mango fruit	Faisalabad	22 (11)	50%	348.0 ± 1360.2	6415	7 (31.8%)
Mango fruit	Sheikhupura	9 (3)	33.3%	42.6 ± 106.1	313	2 (22.2%)
Mango fruit	Multan	15 (8)	53.3%	87.5 ± 166.6	611	5 (33.3%)
Mango fruit	Shorkot	14 (10)	71.4%	254.2 ± 536.9	2030	8 (57.1%)
Mango fruit	Rawalpindi	10 (4)	40%	14.7 ± 34.2	113	1 (10%)
Mango juice	Local markets	40 (30)	75%	24.3 ± 38.1	226	4 (10%)
Mango pulp	Super markets	8 (7)	87.5%	82.3 ± 115.0	301	2 (25%)
Mango jam	General stores	15 (9)	60%	5.0 ± 4.3	13	0 (0%)
TOTAL	--	133 (82)	61.7%	110.9 ± 586.4	6415	29 (21.8%)

**Table 3:** Incidence of patulin (µg/kg) in orange fruits and derived products.

Sample	Sampling Site	n Total (Positive)	Incidence %	Mean ± SD µg/kg	Maximum Value µg/kg	n (%) > 50 µg/kg
Orange fruit	Faisalabad	30 (18)	60%	7.6 ± 7.4	25	0 (0%)
Orange fruit	Sargodha	9 (8)	88.9%	8.1 ± 4.6	15	0 (0%)
Orange fruit	Layyah	14 (4)	28.6%	8.7 ± 19.9	61	1 (7.2%)
Orange fruit	Toba Tek Singh	13 (7)	53.9%	5.1 ± 5.4	15	0 (0%)
Orange fruit	Sahiwal	11 (2)	18.2%	1.6 ± 3.9	12	0 (0%)
Orange juice	Local markets	35 (25)	71.4%	8.3 ± 7.7	31	0 (0%)
Orange pulp	Super markets	10 (6)	60%	6.5 ± 10.9	37	0 (0%)
Orange jam	General stores	19 (4)	21%	1.1 ± 2.2	6	0 (0%)
TOTAL	--	141 (74)	52.5%	6.3 ± 9.1	61	1 (0.7%)
